# Visualization of Coronary Wall Atherosclerosis in Asymptomatic Subjects and Patients with Coronary Artery Disease Using Magnetic Resonance Imaging

**DOI:** 10.1371/journal.pone.0012998

**Published:** 2010-09-29

**Authors:** Suzanne C. Gerretsen, M. Eline Kooi, Alfons G. Kessels, Simon Schalla, Marcus Katoh, Rob J. van der Geest, Warren J. Manning, Johannes Waltenberger, Jos M. A. van Engelshoven, Rene M. Botnar, Tim Leiner

**Affiliations:** 1 Department of Radiology, Maastricht University Medical Center, Maastricht, The Netherlands; 2 Cardiovascular Research Institute Maastricht (CARIM), Maastricht University, Maastricht, The Netherlands; 3 Department of Clinical Epidemiology and Medical Technical Assessment, Maastricht University Medical Center, Maastricht, The Netherlands; 4 Department of Cardiology, Maastricht University Medical Center, Maastricht, The Netherlands; 5 Department of Diagnostic and Interventional Radiology, Saarland University Hospital, Homburg, Germany; 6 Department of Radiology, Division of Image Processing (LKEB), Leiden University Medical Center, Leiden, The Netherlands; 7 Department of Medicine, Cardiovascular Division and Department of Radiology, Beth Israel Deaconess Medical Center and Harvard Medical School, Boston, Massachusetts, United States of America; 8 Imaging Sciences Division, King's College, London, United Kingdom; Universität Würzburg, Germany

## Abstract

**Background:**

Magnetic resonance imaging (MRI) is sensitive to early atherosclerotic changes such as positive remodeling in patients with coronary artery disease (CAD). We assessed prevalence, quality, and extent of coronary atherosclerosis in a group of healthy subjects compared to patients with confirmed CAD.

**Methodology:**

Twenty-two patients with confirmed CAD (15M, 7F, mean age 60.4±10.4 years) and 26 healthy subjects without history of CAD (11M, 15F, mean age 56.1±4.4 years) underwent MRI of the right coronary artery (RCA) and vessel wall (MR-CVW) on a clinical 1.5T MR-scanner. Wall thickness measurements of both groups were compared.

**Principal Findings:**

Stenoses of the RCA (both < and ≥50% on CAG) were present in all patients. In 21/22 patients, stenoses detected at MRI corresponded to stenoses detected with conventional angiography. In 19/26 asymptomatic subjects, there was visible luminal narrowing in the MR luminography images. Fourteen of these subjects demonstrated corresponding increase in vessel wall thickness. In 4/26 asymptomatic subjects, vessel wall thickening without luminal narrowing was present. Maximum and mean wall thicknesses in patients were significantly higher (2.16 vs 1.92 mm, and 1.38 vs 1.22 mm, both p<0.05).

**Conclusions:**

In this cohort of middle-aged individuals, both patients with stable angina and angiographically proven coronary artery disease, as well as age-matched asymptomatic subjects. exhibited coronary vessel wall thickening detectable with MR coronary vessel wall imaging. Maximum and mean wall thicknesses were significantly higher in patients. The vast majority of asymptomatic subjects had either positive remodeling without luminal narrowing, or non-significant stenosis.

**Trial registration:**

ClinicalTrials.gov NCT00456950

## Introduction

Coronary artery disease (CAD) remains the leading cause of mortality and morbidity in the Western World and developing countries despite continued improvements in prevention and early diagnosis[1], [2]. The most frequent cause underlying an acute coronary event is disruption of an atherosclerotic plaque[3], [4]. Rupture-prone plaques are referred to as thin-cap fibroatheroma and characterized pathologically as having a large necrotic core, high macrophage content and a thin, fibrous cap[3], [4].

In asymptomatic individuals acute myocardial infarction and sudden death may be the first clinical manifestation of coronary atherosclerosis[5]. About 50–64% of all sudden cardiac deaths occur without prior recognition of coronary heart disease[1]. Well-established risk factors for coronary atherosclerosis are hypercholesterolaemia, hypertension, cigarette smoking, diabetes mellitus, and systemic inflammation[6], [7]. However, despite their usefulness for global risk assessment, these parameters lack specificity for prediction of individual coronary plaque burden.

Detection of subclinical coronary atherosclerosis in high-risk patients might become an important strategy to prevent clinical coronary heart disease. Compared to other imaging modalities magnetic resonance imaging (MRI) has superior ability to differentiate soft tissues and has shown to facilitate characterization of atherosclerotic plaque components in the aorta and carotid artery by using multisequence MRI[8], [9], [10]. These properties make it uniquely suited for serial non-invasive imaging of the vessel wall, and for monitoring effects of pharmaceuticals on plaque progression or regression. Prior work has demonstrated the capability of MRI to visualize the coronary vessel wall (CVW) with high spatial resolution, as well as vessel wall thickening and positive Glagov-type remodeling in patients[11], [12], [13].

In this study we sought to evaluate the prevalence and extent of coronary atherosclerosis in a cohort of middle-aged subjects without clinical CAD. MR coronary vessel wall (MR-CVW) characteristics were compared to a positive control group of patients with angiographically confirmed CAD.

## Materials and Methods

### Subjects

Between October 2005 and February 2007, 26 middle-aged subjects without previous history or clinical symptoms of CAD and 25 patients with symptoms of stable angina and angiographically confirmed CAD were enrolled. Healthy subjects were recruited via newspaper advertisement. Patients were recruited from the cardiology outpatient clinic of our hospital after having x-ray coronary angiography (CAG). Patients with confirmed CAD underwent MRI prior to percutaneous coronary intervention. Hemodynamically unstable patients or patients with previous interventions in the right coronary artery (RCA) as well as patients with severe arrhythmia or contra-indications for MRI were excluded. Approval of the Maastricht University Medical Center review board and written informed consent of all participants were obtained prior to inclusion.

In addition to registration of demographic data and risk factors, the estimated glomerular filtration rate (eGFR) and PROCAM-score[14] were calculated whenever data were available.

### MR Imaging protocol

All MR studies were performed on a clinical 1.5T system (Intera, Release 11.1 Philips Medical Systems, Best, The Netherlands) using a dedicated 5-element phased-array cardiac radiofrequency (RF) coil. Subjects were examined in the supine position. First, localizer scans were obtained and the subject specific middiastolic trigger delay and acquisition window were determined. These scans were followed by a double oblique oriented three-dimensional (3D) bright blood balanced steady state free precession (bSSFP) coronary MR angiography (CMRA) of the RCA (TR/TE/FA: 6.2/3.1/120°, resolution: 0.98×0.98×3 mm). In the same orientation, a vessel wall scan was acquired (3D FFE, radial k-space sampling, modified double inversion recovery (DIR) prepulse) as previously described[13], [15], [16]. Imaging parameters for the vessel wall scan were: TR: 8 ms, repeated every other heartbeat for good blood suppression, TE: 2.0 ms, Flip Angle: 30°, Field of view: 300×300 mm and matrix: 384×384. Twenty interpolated slices of 1 mm were obtained. The resulting (acquired) spatial resolution was 0.78×0.78×2 mm. Cardiac gating and a 2D navigator beam were used for compensation of cardiac and respiratory motion. This protocol has previously been shown to result in highly reproducible images of the coronary vessel wall in healthy volunteers[16]. No contrast agents or sublingual nitroglycerin were used.

### Evaluation of coronary artery stenosis and plaque burden

Image quality (IQ) of the vessel wall datasets was scored by two experienced, independent blinded observers on a 4 point scale: 1 = well-defined vessel wall borders and high vessel wall-to-background contrast; 2 = some blurring of the vessel wall borders, average to good vessel-wall to background contrast; 3 = major artifacts or severe blurring of the vessel wall borders, low to average vessel wall-to-background contrast, and 4 = vessel wall cannot be identified, very low vessel wall-to-background contrast (modified from Zhang et al. and Spuentrup et al.[17], [18]). An experienced cardiologist blinded to the results of the vessel wall scans assessed the location and degree of a stenosis on the CMRA images. Coronary artery luminograms were classified as negative when there was no visible sign of disease or mild to moderate luminal narrowing (stenosis <50%). Significant disease (stenosis ≥50%) was considered to be present in cases of obvious narrowing of the vessel or marked attenuation of coronary lumen signal[19]. X-ray coronary angiography (CAG) - available in all CAD patients – was visually interpreted by different cardiologists prior to MRI.

Subsequently, an observer blinded to clinical data quantitatively analyzed coronary artery wall images. Minimum, mean and maximum wall thickness were measured on the 3D vessel wall sequence. Lumen diameter was measured on bSSFP images. For measurements, a custom made software package was used (VesselMASS, Leiden University Medical Center, department of Radiology, division Image Processing, Leiden, the Netherlands)[20], adapted for viewing and analysis of longitudinal coronary vessel wall images. For analysis purposes the RCA vessel wall was divided into three segments according to the American Heart Association classification[21].

**Figure 1:**
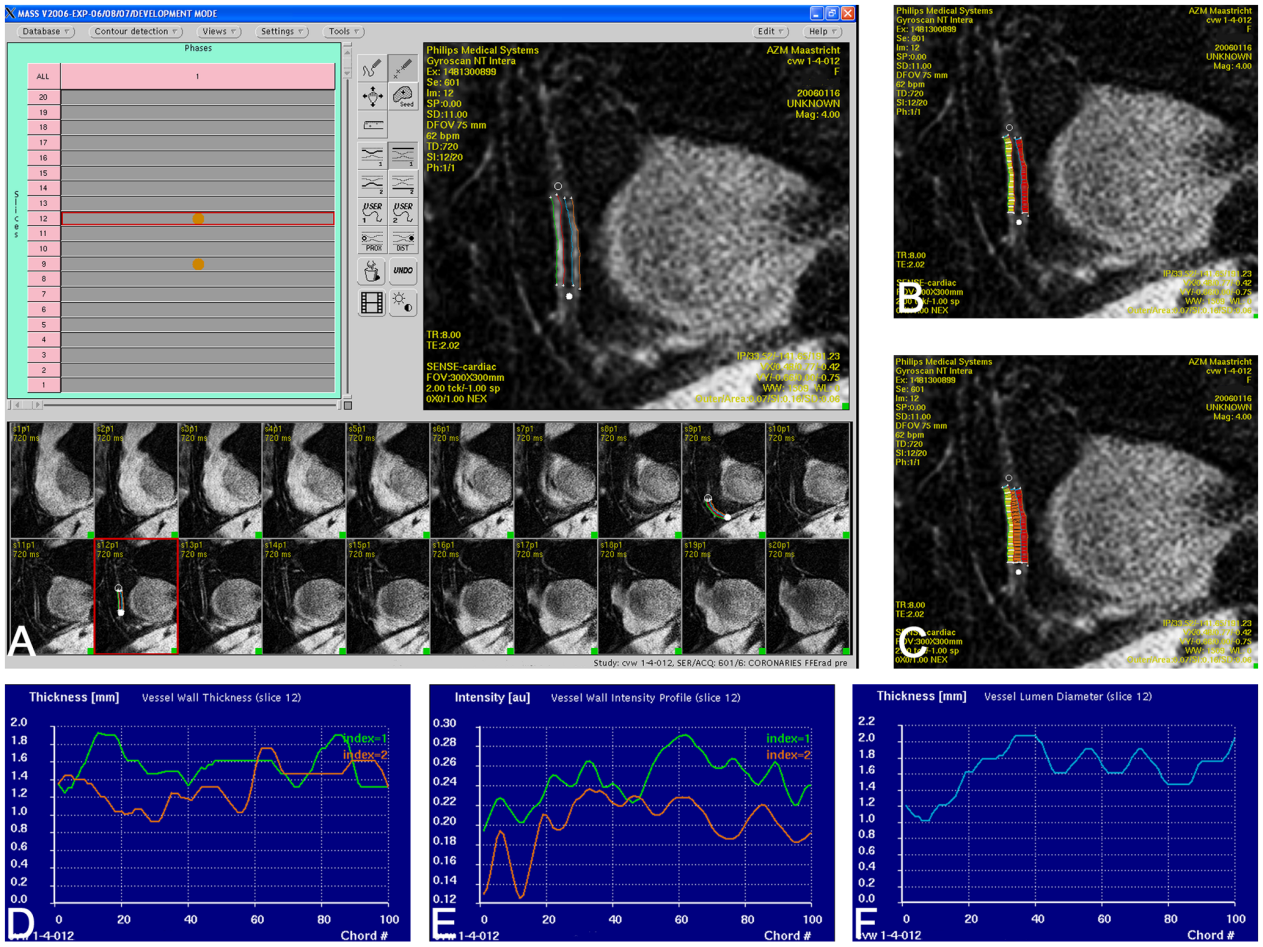
Measurement of vessel wall thickness and signal intensity on source images. The anterior and posterior vessel walls are delineated (A). In each segment 100 measurements are made of the thickness and signal intensity of the proximal and distal RCA vessel wall and the diameter of the lumen (B,C). The custom made software program calculates wall thickness, signal intensity, and lumen diameter (D–F) over the entire length of the measured segment.

To prevent errors in wall thickness and lumen diameter measurements due to reconstruction errors, only source images were used for measurements. In source images where the vessel wall was visible, the inner and outer borders of the anterior and posterior vessel wall were manually delineated (Figure 1). Each resulting region of interest (ROI) was automatically divided in 100 subsegments for quantitative analysis of wall thickness. Because measurements in adjacent subsegments are not independent due to partial volume effects we corrected for the length of the measured segment and spatial resolution by only using subsegments from separate voxels and discarding measurements in between. The number of included segments equals the number of voxels in the ROI, which was calculated by dividing the length of the ROI by the in-plane spatial resolution. Coronary plaque burden was quantified as the mean and maximum RCA vessel wall thickness along the proximal two vessel segments[19]. In addition to measuring wall thickness, a normalized wall index was calculated (100*(diameter lumen divided by the sum of anterior and posterior wall thickness)) to get a better quantitative impression about the degree of positive remodeling.

Based on both CMRA and MR-CVW, areas could be classified as diseased or not-diseased according to the following criteria: ROIs with vessel wall thickening were classified as diseased. This could either be in areas of stenosis (combination of luminal narrowing with vessel wall thickening) or in areas with positive remodeling (wall thickening without luminal narrowing. ROIs with normal lumen diameter compared to the rest of the vessel and with a vessel wall that had no areas of focal wall thickening or focal high signal intensity, were classified as “not diseased”. Since calcified plaques demonstrate low signal intensity on all MR sequences and can therefore be difficult to visualize, ROIs with obvious luminal narrowing but without visible vessel wall thickening were interpreted as diseased with (heavily) calcified plaque if overall image quality of the vessel wall scan was good and if the vessel wall and lumen could be distinguished in other segments of the vessel.

### Statistical analysis

Statistical analysis was performed using SPSS 11.5 (SPSS Inc., Chicago, IL, USA). Differences in IQ between the patient and control group were compared using a Wilcoxon rank-sum test. Minimum, maximum and mean wall thickness and the normalized wall index in both groups were compared using an unpaired student t-test. Differences between men and women in both groups were analyzed using a Wilcoxon rank-sum test.

In addition, we pooled the results by testing the global hypothesis that all results of the measurements (minimum, maximum and mean values) of vessel wall thickness were equal using the ordinary least square test[22], [23]. Significance was assumed at two-tailed *p* values of less than 0.05.

## Results

### Subject characteristics

All 51 subjects underwent coronary MRI without complications. In three patients, the coronary vessel wall sequence could not be performed due to severe back pain (1 patient) and software problems (2 patients). This resulted in evaluable vessel wall scans in 26 asymptomatic subjects (11 M, mean age 56±4 yrs) and 22 patients (15 M, mean age 60±10 yrs).

Clinical characteristics of this study cohort are listed in table 1. There was a significant difference in subjects' weight and the presence of hypercholesterolemia and hypertension between both groups. In the patient group, there was a trend towards higher prevalence of males and higher age, higher incidence of diabetes, and higher PROCAM score. In patients (n = 12) in whom a PROCAM score could be calculated, a 10-year risk for acute coronary events of 12.0% (±8.1, range: 1.3–25.1%) was present, versus 4.8% (±2.4, range: 1.9–8%) in control subjects (n = 6). Linear regression showed a weak correlation between eGFR and mean wall thickness (R: 0.54, p<0.01). In subjects with low eGFR decreased vessel wall thickness was found.

**Table 1:** Subject characteristics.

	Patients(N = 22)	Asymptomatic subjects(N = 26)	P
characteristics			
Gender, male n (%)	15 (68)	11 (42)	0.08
Age, y (±SD)	60.41 (10.35)	56.08 (4.43)	0.08
Weight, kg (±SD)	81.27 (15.59)	71.35 (13.73)	0.02
Systolic blood pressure, mm Hg (±SD)	137 (14.16)	133 (16.38)	0.39
Diastolic blood pressure, mm Hg (±SD)	80 (9.28)	79 (11.46)	0.87
Diabetes, n (%)	3 (13.6)	0 (0)	0.08
Current smoker, n (%)	3 (13.6)	5 (19.2)	0.61
Hypertension, n (%)	13 (59.1)	1 (3.8)	<0.01
Hypercholesterolemia, n (%)	11 (50)	2 (7.7)	0.01
eGFR (±SD) mL*min^−2^ *1.73 m^−2^	88.61 (27.5) (n = 22)	76.01 (24.2) (n = 9)	0.24
PROCAM-score (±SD)†	44.6 (9.7) (n = 12)	36.0 (6.0) (n = 6)	0.07

*eGFR: estimated Glomerular Filtration Rate.

† PROCAM-score: based on the PROspective CArdiovascular Muenster study-scoring scheme.

### Coronary artery atherosclerosis

The prevalence of coronary stenoses as identified by CMRA in the 48 subjects with complete scans is listed in Table 2. Significant coronary artery stenoses (≥50%) were found in 17 patients on CMRA, but only in 16 patients on CAG. In 19/26 asymptomatic subjects (73%), there was evidence of at least some luminal narrowing on CMRA. Nine of these 19 asymptomatic subjects had a significant stenosis (≥50%). In the patient group, the degree of stenosis as estimated on CMRA (< or ≥ than 50%) was compared to the results of CAG. In 21 out of 22 patients (95%), location of stenoses detected on CMRA corresponded to stenoses detected with CAG, although in some segments there was overestimation of the degree of stenosis on CMRA. Agreement between CAG and CMRA for detection of lesions is demonstrated in table 3. Sensitivity and specificity were 100% and 83%, respectively.

**Table 2:** Stenoses on coronary MRA.

	Patients(N = 22)	AsymptomaticSubjects (N = 26)
No stenoses	0 (0)	7 (27)
<50% stenoses	5(23)	10 (38)
≥50% stenoses	17 (77)	9 (35)

Prevalence of stenoses (number (%)) in the right coronary artery as seen with coronary MRA.

**Table 3:** Comparison between CMRA and CAG on segment level.

		CAG		Non-evaluable segments
		0–50%	≥50%	
CMRA proximal	<50%≥50%	102	010	0
CMRA mid (*)	<50%≥50%	103	08	1 (CAG)
CMRA distal	<50%	10	0	4 (CMRA)1 (CAG)
	≥50%	5	2	

Agreement between coronary MRA (CMRA) and X-ray coronary angiography (CAG) on degree of stenoses in the right coronary artery in patients with coronary artery disease (N = 22) in the proximal, mid and distal RCA. Values represent number of segments.

Lumen diameter as measured on MR-CVW, was slightly smaller compared to diameters measured on CMRA. In patients, lumen diameter on CMRA was 1.86±0.46 mm, versus 1.66±0.53 mm on MR-CVW (p = 0.02). In asymptomatic subjects, lumen diameter was 2.01±0.55 on CMRA and 1.64±0.63 on MR-CVW (p<0.01). The slight difference in in-plane spatial resolution between both techniques (0.99 mm on CMRA, 0.78 mm on MR-CVW), can theoretically lead to overestimation of the lumen diameter on CMRA, but also overestimation of coronary stenoses, or underestimation of lumen diameter on MR-CVW. However, on MR-CVW partial volume may play a more important role since the lumen is surrounded by vessel wall. It is more probable that lumen measurements are more accurate on CMRA, although a slight influence of the signal of the vessel wall on diameter measurements on CMRA cannot be ruled out completely. In theory however, we expect that this effect is minimal due to the technique used for suppression of myocardial signal (T2prep). Since the vessel wall is mainly composed of smooth muscle cells, collagen and elastin, the T2 is expected to be similar to the T2 of myocardium. Because of signal suppression due to T2prep, only little signal contribution of the vessel wall can be expected.

There was a trend towards smaller minimum lumen diameter as measured on the luminography sequence in the patient group (1.06±0.58 in patients vs 1.35±0.53 mm in asymptomatic subjects, p = 0.08). However, no significant differences could be demonstrated between mean (1.86±0.46 mm in patients, 2.0±0.55 mm in asymptomatic subjects, p = 0.35) and maximum (2.91±0.57 mm in patients, 2.73±0.70 mm in subjects, p = 0.35) luminal diameters of the two groups (Table 4).

**Table 4:** Vessel wall thickness and lumen diameter measurements.

	Patients(N = 22)	Asymptomatic subjects (N = 26)	p
*Vessel wall:*			
Minimal vessel wall thickness (mm) (±SD)	0.71 (0.19)	0.65 (0.16)	0.269
Maximal vessel wall thickness (mm) (±SD)	2.16 (0.37)	1.92 (0.44)	0.048
Mean vessel wall thickness (mm) (±SD)	1.38 (0.18)	1.22 (0.22)	0.011
Overall (standardized) wall thickness (±SD)	0.87 (2.25)	−0.74 (2.57)	0.026
*Lumen CMRA*			
Minimal lumen diameter (mm) (±SD)	1.06 (0.58)	1.35 (0.53)	0.080
Maximal lumen diameter (mm) (±SD)	2.91 (0.57)	2.73 (0.70)	0.354
Mean lumen diameter (mm) (±SD)	1.86 (0.46)	2.01 (0.55)	0.346
*Lumen MR-CVW*			
Minimal lumen diameter (mm) (±SD)	0.91 (0.58)	1.02 (0.53)	0.475
Maximal lumen diameter (mm) (±SD)	2.57 (0.66)	2.39 (0.81)	0.396
Mean lumen diameter (mm) (±SD)	1.66 (0.53)	1.64 (0.63)	0.930
*Normalized wall index (%),* (±SD)	68.2 (17.0)	82.8 (20.7)	0.012

Vessel wall thickness and lumen diameter measurements (±SD) measured on both CMRA and MR-CVW in patients and healthy volunteers. Normalized wall index was calculated as a value to represent positive remodeling according to the following formula: 100*(lumen diameter/sum of anterior and posterior wall thickness).

IQ of the vessel wall scans was good in the majority of cases with a mean score of 1.42±0.58 for all subjects. There was no significant difference in IQ between patients and asymptomatic subjects (1.32±0.57 vs 1.5±0.58 for observer 1, p = 0.21, 1.42±0.58 vs 1.41±0.59 for observer 2, p = 0.9).

**Figure 2:**
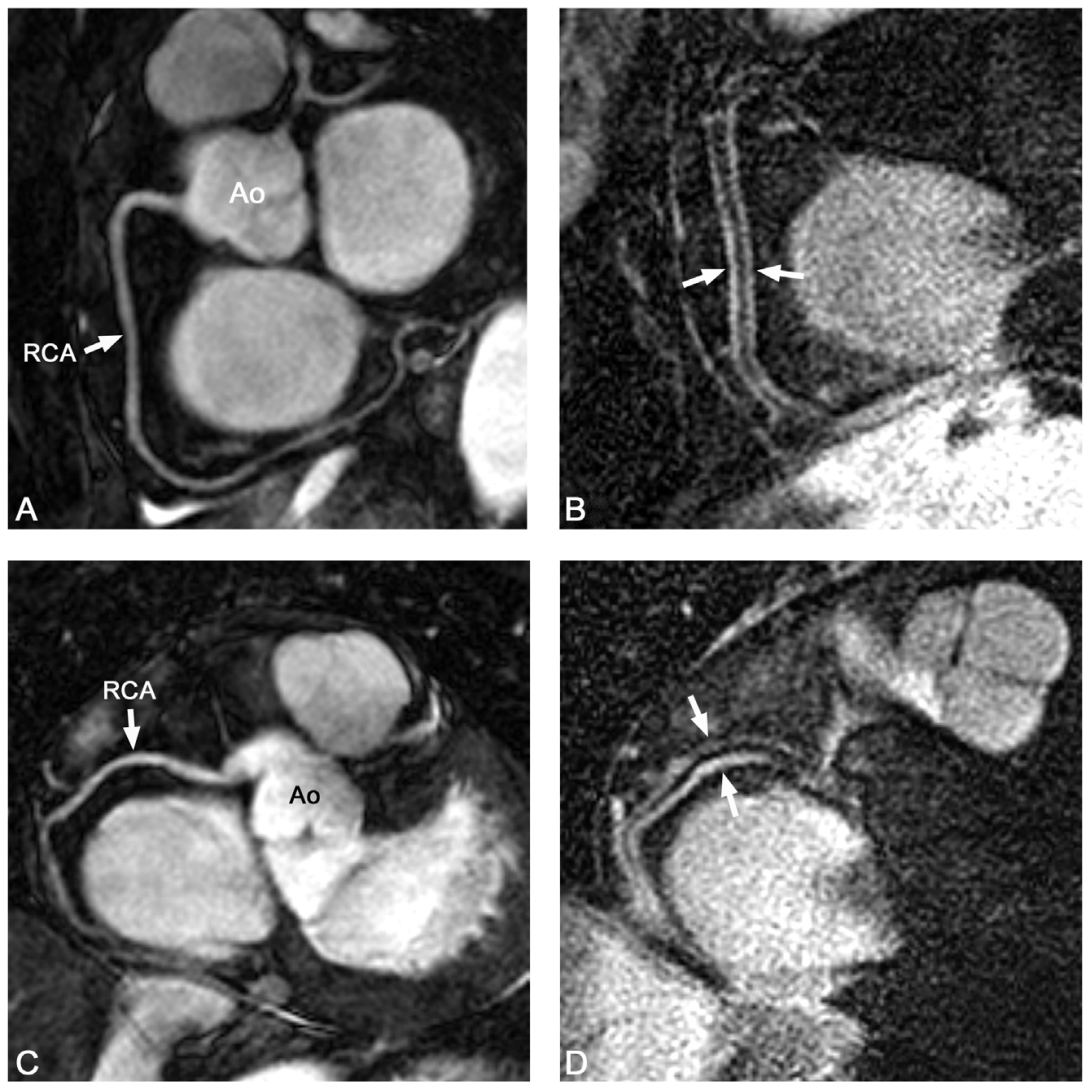
MRA and MR-CVW in asymptomatic subjects. Balanced steady state free precession (bSSFP) coronary MRA (A,C) and corresponding vessel wall scans (B,D) of the right coronary artery (RCA) in asymptomatic subjects. A and B represent the RCA of a 62 y/o female (A,B). There is a thin vessel wall with uniform signal intensity (arrows in B). In C and D, the RCA of a 54 y/o female is shown. In this subject, the posterior proximal vessel wall is thicker and has higher signal intensity compared to the anterior vessel wall (arrows in D), indicating the presence of positive remodeling. Ao indicates aorta.

**Figure 3:**
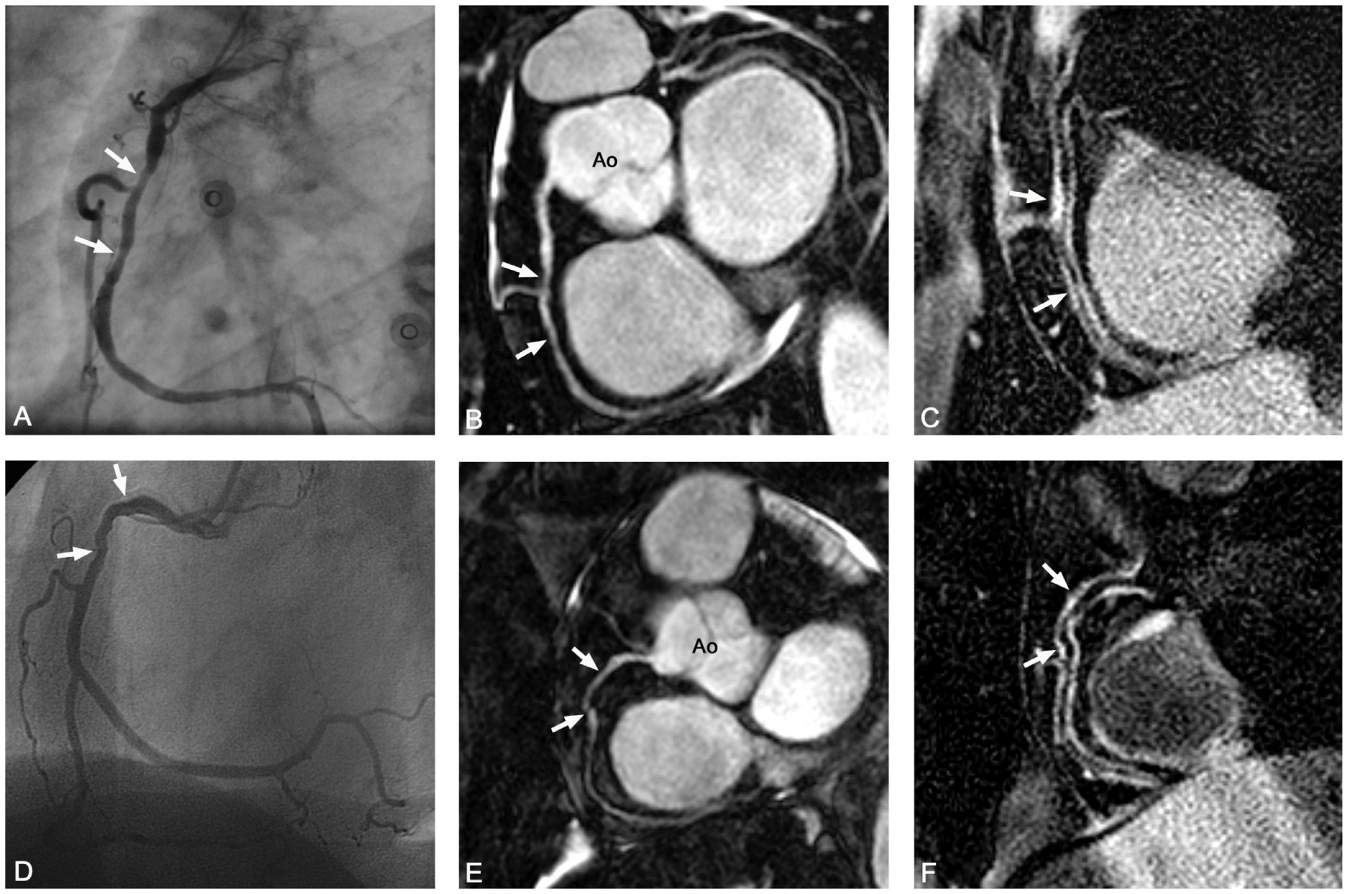
MRA and MR-CVW in patients with stable angina. X-ray coronary angiography (A,D), balanced steady state free precession (bSSFP) coronary MRA (B,E) and corresponding vessel wall scans (C,F) of the right coronary artery (RCA) in two patients with stable angina. In a 40 y/o male (A–C), vessel wall thickness and signal intensity are increased in areas of stenosis and wall irregularities (arrows). In a 67 y/o female (D–F) the culprit lesion was located in the left anterior descending artery (not imaged). However, the proximal RCA demonstrated wall focal luminal irregularities (arrows in D,E). In F, corresponding focal heterogeneity in wall thickness and signal intensity of the entire vessel wall are clearly visible. Ao indicates aorta.

Various degrees of non-uniform vessel wall thickening were seen on the MR-CVW scans in patients and asymptomatic subjects (Figures 2 and 3). Quantitative results are summarized in Table 4. Maximum and mean wall thicknesses were significantly larger in the patient group. The normalized wall index was higher in asymptomatic subjects when compared to patients (82.8 ± 20.7 versus 68.2 ± 17.0, p = 0.012), representing an increased wall thickness in all patients and asymptomatic subjects but a relatively thicker vessel wall compared to lumen diameter in patients. Pooled results of wall thickness also demonstrated a significant difference between the groups (p = 0.03).

Overall, there was good agreement between luminal narrowing at CMRA and increased wall thickness at MR-CVW in all patients. Agreement between CMRA and MR-CVW in asymptomatic subjects is demonstrated in Table 5. There were 5 asymptomatic subjects with stenoses without wall thickening: in three of these subjects, the area of stenosis was not well visualized on the vessel wall scan, in one subject vessel wall and lumen could hardly be distinguished and in one subject the location of the vessel wall thickening did not correspond to the area of stenosis on CMRA.

**Table 5:** Agreement between MR coronary vessel wall imaging (MR-CVW) and coronary magnetic resonance imaging (CMRA) for detection of stenoses and vessel wall thickening in asymptomatic volunteers.

		CMRA	
		No stenoses	Stenoses
MR-CVW	No Wall thickening	3	5
	Wall thickening	4	14

Values represent number of subjects.

The groups were age-matched, but not matched for gender. There was a trend towards a higher prevalence of women in the group of healthy volunteers (Table 1). In patients, there were no significant differences between men and women. However, in the group of healthy volunteers, there was a significant difference in mean vessel wall thickness, mean luminal diameter on CMRA, mean luminal diameter on MR-CVW and a trend towards difference in normalized wall index (Table 6).

**Table 6:** Gender related differences in the group of patients (n = 22) and the group of healthy volunteers (n = 26).

	Men	Women	p
*Patients (15M, 7F)*			
Mean vessel wall thickness (mm) (±SD)	1.41 (0.18)	1.32 (0.18)	0.341
Lumen diameter on CMRA (mm) (±SD)	1.90 (0.38)	1.79 (0.61)	0.307
Lumen diameter on MR-CVW (mm) (±SD)	1.66 (0.49)	1.65 (0.63)	0.805
Normalized wall index (%), (±SD)	67.8 (13.0)	69.1 (24.9)	0.916
*Healthy subjects (11M, 15F)*			
Mean vessel wall thickness (mm) (±SD)	1.34 (0.24)	1.14 (0.18)	0.046
Lumen diameter on CMRA (mm) (±SD)	2.43 (0.57)	1.69 (0.26)	0.001
Lumen diameter on MR-CVW (mm) (±SD)	2.04 (0.75)	1.36 (0.31)	0.005
Normalized wall index (%), (±SD)	94.0 (26.6)	74.6 (9.3)	0.092

## Discussion

This is one of the few studies to use non-invasive MRI to assess coronary artery plaque burden in an asymptomatic population-based cohort of adults using magnetic resonance CVW imaging. The most significant finding of this study is the high prevalence of coronary wall plaque in asymptomatic adults free of known CAD. Four out of 26 healthy subjects exhibited evidence of positive remodeling without luminal narrowing and the majority of subjects (19/26) exhibited evidence of luminal narrowing at CMRA, in some cases even exceeding 50%. As expected, the burden of coronary atherosclerosis was higher in the positive control group, which consisted of patients with angiographically confirmed CAD. Despite the lack of a gold standard for visualization of the coronary vessel wall (i.e. intravascular ultrasound), these data suggest that MRI may help to non-invasively identify not only late stages of atherosclerosis but also the early manifestations of atherosclerotic disease in the coronary vessel wall.

Our results corroborate the findings of Kim et al, who investigated subjects with longstanding diabetes type 1 but without cardiovascular symptoms[19]. In these asymptomatic patients, only 10% of patients with nephropathy and 0% of patients with normoalbuminuria had evidence of coronary stenoses although the prevalence of visually identifiable coronary plaque in the RCA was higher (76% in patients with nephropathy versus 15% in patients with normoalbuminuria). The prevalence of vessel wall thickening in the RCA is in the same range as in our study group, however, we found more cases of luminal narrowing, probably reflecting differences in study populations. Although diabetic subjects tend to have more atherosclerosis, our asymptomatic subjects were older than the subjects with diabetic nephropathy from the study by Kim et al (mean age of 56 vs 48 years). Additionally, MRI is known to overestimate stenosis and possible slight differences in spatial resolution between the used techniques can contribute to differences in assessment of stenosis. Tuzcu et al[24] studied a population of 262 subjects and found vessel wall thickening of more than 0.5 mm on intravascular ultrasonography (IVUS) in >70% of asymptomatic subjects by the age of 40, while only 8% of X-ray coronary angiograms of the entire cohort demonstrated mild luminal irregularities. While the study by Tuzcu once again underscores that early vessel wall abnormalities go undetected at coronary angiography, there was a large difference in prevalence of luminal irregularities compared to our study. The fact that our study demonstrated luminal irregularities on CMRA in >70% of subjects compared to 8% in the study by Tuzcu et al. can be explained by the better spatial resolution of X-ray coronary angiography compared to CMRA, the fact that flow disturbances on CMRA can lead to overestimation of the degree of stenoses, and the difference in age and case-mix between the populations.

A recent publication from the Multi-Ethnic Study of Atherosclerosis (MESA) also found positive remodeling in a large cohort of 179 subjects without known coronary artery disease[25]. In contrast to our study, the MESA study did not find any significant stenoses (≥50%), despite the use of similar spatial resolution as well as an identical technique for suppressing blood flow[25]. Axial images of all three coronary arteries (LM, RCA and LAD) were acquired, but due to time constrains this was limited to the proximal parts of the vessels of interest, with only one slice in the LM and 3 slices in the RCA and LAD. Axial imaging of the coronary vessel wall is very time consuming, and the longitudinal technique that we used offers an overview of the vessel wall in a much larger part of the coronary artery of interest. The difference in acquisition approach, especially the chosen imaging plane and resulting differences in partial volume artefacts, between our study and the MESA study is one of the likely explanations for the discordant results.

Clinical CAD has a long asymptomatic period during which biological risk factors interact with genetic and environmental influences to initiate and promote the development of coronary atherosclerotic plaque[26], [27]. Once established, atherosclerosis can exist in a subclinical state characterized by the presence of plaque but an absence of clinical signs and symptoms. Acute clinical manifestations of coronary atherosclerosis such as unstable angina and myocardial infarction are usually the consequence of acute rupture of the atherosclerotic plaque, leading to exposure of thrombogenic components to the bloodstream, with superimposed thrombus formation[28]. Although traditional risk factors and risk scores such as the Framingham[7], [29] and PROCAM[14] scores are accurate at predicting risk to experience a cardiovascular event in the near future for groups of patients, they often lack discriminative power to characterize individual subjects risk. Approximately 40% of the adult population in the U.S. and Western Europe are at intermediate (0.5–2.0%) and 10% at high risk (>15%) for developing an acute coronary event over the next decade[30]. Therefore, further risk stratification of individual patients with CAD using novel biochemical markers as well as noninvasive imaging techniques may be important. Our study was motivated by the paucity of data on the prevalence and characteristics of early CAD and coronary atherosclerotic plaque burden at MRI in an unselected cohort of asymptomatic middle aged adults free of known CAD at baseline. However, it is known that the measured degree of coronary stenosis that was taken as significant in this study (i.e. ≥50%) often is not associated with significant impairment in coronary blood flow. This might be the reason why none of the control subjects with stenoses ≥50% at MRI were symptomatic. An alternative explanation could be that with MRI luminal irregularities are erroneously overestimated[31], [32], [33], which is one of the shortcomings of MRI in comparison to conventional X ray angiography.

Whether MR-CVW confers additional information over more established non-invasive imaging techniques such as calcium scoring[34], [35] and multislice computed tomography (MSCT)[36], [37] is unknown. Spatial resolution of CT is better. However, in comparison to epicardial coronary artery calcium scoring, MRI can detect earlier changes such as outward remodeling. Outward remodeling has been shown to be associated with plaques that have a higher relative content of fibrofatty components as opposed to calcification[38], as well as with plaques that ultimately exhibit an unstable clinical presentation[39]. The excellent soft-tissue resolution of MRI enables better characterization of plaque components than CT. Furthermore, no contrast agents are needed to visualize the coronary vessel wall with MRI, which is beneficial in patients with renal failure or allergies. An additional advantage of MRI is that it does not require radiation exposure, an issue that takes on considerable importance when weighing risks and benefits in asymptomatic subjects, and when follow-up studies are desired[40].

Limitations of this study should be considered. Due to ethical considerations, no comparison to a standard of reference was obtained in asymptomatic volunteers and therefore, there is no certainty about actual prevalence of abnormalities in this group. Furthermore, the groups were matched for age but not for gender. It is known that there are gender related differences in coronary artery disease. In general, women have a less typical clinical presentation and often have fewer angiographic high-risk features[41], [42]. In our group of asymptomatic volunteers, there were several significant differences between men and women. Women had thinner vessel walls and smaller lumen diameter, however, the lower normalized wall index in women indicates a relatively thick vessel wall compared to lumen diameter, which could be related to more positive remodeling. In patients, these differences between men and women were not found. Our results are supported by the study by Miao et al. who also found gender related differences in lumen size, vessel wall area and mean vessel wall thickness in a large cohort of asymptomatic men and women who underwent MRI of the coronary vessel wall [25]. However, in both men and women, they found a similar, significant, relationship between vessel wall thickness and outer lumen area or lumen area, both measures for the degree of positive remodeling. In the study by Miao et al, there was no comparison between patients with CAD and healthy subjects. Due to the difference in the amount of women between both groups in our study, future studies should not only match for age but also for gender to further study possible gender-related differences.

Currently, there are still some limitations in scan technique for coronary vessel wall imaging. With currently used in-plane spatial resolution of 0.78 mm, detection of small variations in wall thickness due to partial volume effects are not yet possible. Additionally, due to the current spatial resolution, it can be expected that partial volume effects account for overestimation of coronary vessel wall thickness. Other factors that lead to overestimation of wall thickness are curvature of the vessel and out of plane orientation of the vessel. However, these factors play a more important role with multiplanar reformatting, and to minimize errors due to these factors, measurements were performed on source images.

Furthermore, because the obvious long-term goal of MR-CVW imaging is not only measurement of plaque size but also composition, increased spatial resolution is needed to characterize plaque components. This is important, as it is known that a large lipid-rich necrotic core and a thin fibrous cap portend a poor prognosis. First attempts at characterization of coronary plaques using contrast-enhanced MR-CVW imaging have been made by Maintz et al[43] and Yeon et al[44], who demonstrated that use of contrast-enhanced MR CVW imaging allowed identification of areas of delayed enhancement that correlated with severity of atherosclerosis by MSCT and quantitative X-ray angiography. Also, whether therapeutic intervention guided by imaging modalities will influence clinical event rates remains an important focus of research. Imaging cannot be regarded as an isolated tool to determine prognosis. However, it is highly likely that imaging will only confer additional information in selected subgroups of subjects in combination with traditional risk factors and (novel) serological markers. This concept is also known as the ‘vulnerable patient’[45]. Further limitations include the relatively low sample size and, because of an attempt to limit total scantime in our subjects, analysis of coronary plaque burden was restricted to the RCA.

In conclusion, although there was low prevalence of significant coronary stenoses, we found coronary atherosclerotic plaque in a large proportion of asymptomatic middle-aged subjects at MR CVW imaging. As expected, there were less stenoses and asymptomatic subjects exhibited lower mean coronary vessel wall thickness compared to a control group of patients with angiographically proven coronary artery disease. Because the time-course from subclinical disease to clinical events is highly variable, it is important that future studies focus on the association between MR CVW evidence of disease and clinically manifest incident CAD.
